# Clear Aligners in Patients with Amelogenesis and Dentinogenesis Imperfecta

**DOI:** 10.1155/2021/7343094

**Published:** 2021-12-23

**Authors:** Nozha M. Sawan

**Affiliations:** Department of Preventive Dental Sciences, College of Dentistry, Princess Nourah Bint Abdulrahman University, Riyadh, Saudi Arabia

## Abstract

Dentinogenesis imperfecta (DI) and amelogenesis imperfecta (AI) are hereditary abnormalities of dental hard tissues. Dental abnormalities may also be accompanied by symptoms of disorders such as osteogenesis imperfecta. AI and DI have a significant burden on socializing, function, and comfort; therefore, frequent screening and accurate diagnosis is the cornerstone of managing such conditions. Both AI and DI could be treated with many strategies, including restorative, prosthetic, periodontal, surgical, and orthodontics treatment. The interdisciplinary combination of orthodontic, prosthodontic, and periodontic treatment has been proven to improve the prognosis of AI and DI. Regarding orthodontic treatment, the most difficult element of orthodontic therapy may be maintaining a high level of motivation for what might be a prolonged form of treatment spanning several years. There are many forms of orthodontic management for AI and DI, including removable appliances, functional appliances, and fixed appliances. Clear aligner therapy (CAT) contains a broad range of equipment that works in different ways, has different construction processes, and is compatible with different malocclusion procedures. The application of CAT in patients with AI and DI is favorable over the fixed applicants. However, the available evidence regarding the application of CAT in AI is weak and heterogeneous. In this review, we discussed the current evidence regarding the application of clear CAT in patients with AI and DI.

## 1. Introduction

Mineralization deficits in dental hard tissues may affect dentin and enamel. Molar-incisor-hypomineralization (MIH) is characterized by minor teeth alterations with an estimated prevalence of up to 25%, depending on the ethnicity [1]. It has been stated that the major risk factors for MIH include oxygen deprivation, mineral deficiency, juvenile infectious disorders, and chronic obstructive pulmonary disease [2]. The association between MIH and enamel alteration was reported, including hypomineralization and hypoplasia of dentin and enamel. The first permanent molars and incisors are frequently the most affected teeth. Many serious dental diseases are hereditary, i.e., inherited in nature [3]. Dentinogenesis imperfecta (DI) and amelogenesis imperfecta (AI) are hereditary abnormalities of dental hard tissues. Dental abnormalities may also be accompanied by symptoms of disorders such as osteogenesis imperfecta [4, 5]. In addition to enamel defects, solitary affection of dentin is possible. Modifications might vary from mild hypomineralization with moderate discoloration to hypoplastic changes with significant loss of tooth hard tissues [6, 7]. The handling of these alterations must be rapid and accurate. Treatment options include restorative, prosthetic, periodontal, surgical, and orthodontic approaches [8–11]. This review aimed to discuss the current evidence regarding the application of clear aligners treatment (CAT) in patients with AI and DI.

## 2. Amelogenesis Imperfecta (AI)

**Figure 1:**
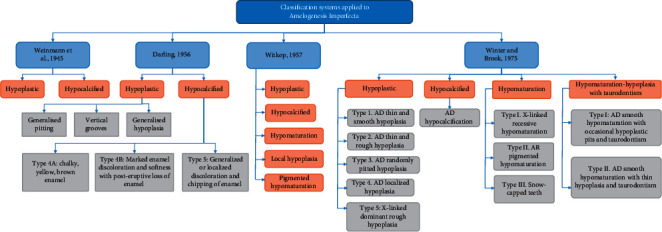
Amelogenesis imperfecta classifications.

AI refers to a group of inherited developmental disorders that alter the structure and clinical appearance of dental enamel in nearly all primary and secondary dentition teeth, as well as other abnormalities in intraoral and extraoral tissues [12]. According to the population investigated, the global prevalence of AI is around <1 in 200 [13]. It can occur alone or as part of a syndrome with additional problems [14, 15]. AI occurs in multiple inheritance patterns, including sex-linked, autosomal recessive, autosomal dominant, and sporadic [16]. AI has been associated with mutations in the amelogenin gene, AMELX, in families with an X-linked variation. The pathophysiology of AI's dominant variations has been connected to the ENAM and FAM83H genes [17–21].  Figure 1 summarizes the classification of AI. In individuals with confirmed consanguinity, autosomal recessive AI has been observed [6].

Patients with hypoplastic AI have thin enamel and a transparent appearance [22]. While enamel thickness is normal in hypocalcified AI, there is a defect in the enamel categorization with weak structure and discolored teeth [13]. In patients with hypomaturated AI, the enamel is characterized by a mottled appearance, and the tooth is vulnerable to wear [23]. Family history, pedigree mapping, and careful clinical observation are all used to diagnose AI. Early and robust preventive and restorative care can control the disease and its effects on socialization and functioning as well as on pain [24]. By inserting prepared metal crowns on the posterior teeth during childhood, the primary dentition can be retained. Long-term maintenance is accomplished by the use of crowns and plastic restorations [25].

## 3. Dentinogenesis Imperfecta (DI)

Dentinal deficiencies are mostly caused by hereditary factors, although they can also be caused by environmental or systemic disorders that affect calcium metabolism or calcification [26]. DI is a genetic condition that predominantly affects the development of dentin [27]. In humans, DI is one of the most frequent autosomal dominant traits. According to the literature, DI has been classified into three clinical entities: type I (DI-I), type II (DI-II), and type III (DI-III). DI-I has 1 in 20,000 birth rate, whereas DI-II and DI-III have 1 in 8000 birth rate [28–31]. DI-I is seen in 20–40% of individuals who have osteogenesis imperfecta. DI-I is defined as teeth with an amber translucency and damaged enamel [32]. DI-II is characterized by opalescent dentin, which has more clinical variability than type I and affects both dentitions equally [33]. Multiple pulp exposures may be seen in deciduous teeth in DI-III [34]. Teeth shape and coloration appear to be more varied than in DI-I and DI-II. Mutations in the dentin sialophosphoprotein gene (DSPP) on chromosome 4q21 have been linked to DI and another dentinal abnormality known as dentin dysplasia (DD) [35]. DI affects both the primary and permanent dentition, and men and women are equally affected. DI's appearance can be described in various ways, including amber-brown, grey-blue, and opalescent, which refers to the appearance of the teeth under transillumination [36]. The primary dentition has a more striking appearance than the secondary dentition, which seems to be less impacted.

## 4. Management of AI and DI

There are numerous treatment options for AI and DI, as discussed previously in this article. These include restorative and prosthetic procedures as well as periodontal and surgical procedures. Only a few studies have shown that direct composite restorations are effective in patients with AI [37–41]. In childhood, it is mostly utilized to gain some time before completing the ultimate repair at adulthood. The longevity of dental restorations is substantially reduced in patients with AI due to the severity of the disease [42]. Recurrent caries was the most prevalent reason for restoration failure in individuals with AI, while the fracture of the restoration or of the tooth was the most common reason in those without AI [43, 44]. In most cases of DI, bonding to the defective dentin has been shown to be effective; however, intracoronal restorations are contraindicated due to the risk of enamel fracture [45].

In terms of prosthetic treatment, many anterior and posterior teeth require extensive coronal restorations [46, 47]. Another option for repairing damaged teeth is porcelain fused to metal (PFM) crowns and bridges. Most recently, all-ceramic crowns have been shown to be effective [48]. Composite crowns, veneers, and even stainless steel crowns may be useful depending on the patient's age [49]. Periodontal therapy requires intensive care and good oral hygiene to protect dental hard tissues and restorations from deteriorating further from caries [50]. Since the teeth damaged by AI and DI are more sensitive, patients are more likely to neglect their dental health, leading to gingivitis [37, 51]. Due to the higher plaque formation caused by rough tooth surfaces, persistent gingivitis and marginal periodontitis are also noticeable, especially in hypoplastic types of AI [52]. As a result, improved oral hygiene and gingivitis prevention are crucial for achieving good restoration lifespan and treatment outcomes. The most common periodontal treatments include scaling and root planning [53].

The most serious orthodontic issue in patients with AI is anterior open bite (AOB), particularly in those with hypoplastic and hypocalcified types [54]. Because teeth are more sensitive to hot and cold due to a lack of enamel and excessive tooth wear, the tongue's malposition because of the changed vertical relationship may prevent vertical malocclusion from being compensated by vertical alveolar growth, allowing the AOB to continue [55]. Since AOB has such a high recurrence rate, orthodontic surgery must be done only if the existing craniofacial anomalies are extremely serious [56].

## 5. Orthodontic Management

For a patient with AI and DI, the most difficult element of orthodontic therapy may be maintaining a high level of motivation for what might be a prolonged form of treatment spanning several years. To be effective, treatment must be broken down into manageable segments with clearly stated goals that the patient can engage with. There are many forms of orthodontic management for AI and DI, including removable appliances, functional appliances, and fixed appliances [57]. Removable appliances can resolve many issues with fixed appliances. Due to the reduction in the crown height and absence of undercuts, the retentive aspect must be carefully considered [58]. Aesthetics should be considered and enhanced wherever possible in order to increase patient compliance [59]. Regarding functional appliances, it is mainly used in cases of class II malocclusion. Subsequent restorations generally require vertical clearance and an open bite; the vertical element must be carefully managed [60]. In terms of fixed appliances, when the appliance is implanted, it is important to schedule an appointment so that the patient may return to the operation if the device debonds. In addition, due to the patient's sensitivity, a staged bonding process is typically necessary [57].

## 6. Clear Aligners in Orthodontic Treatment

Clear aligner therapy (CAT) contains a broad range of equipment that works in different ways, has different construction processes, and is compatible with different malocclusion procedures [61]. All utilize transparent thermoplastic aligners to protect many or all of the teeth, but there are substantial variations that impact each system's capacity to manage a broad range of orthodontic disorders [62]. Initially, CAT was only used to correct minor tooth position abnormalities. Recently, some aligner systems have been specifically designed to address minor positional abnormalities, while others claim to be able to cure complicated malocclusion [61]. Nonetheless, several CAT devices are available for purchase by the general public, and some (such as Crystal Braces and Smile Care Club) do not even require the participation of a dentist at any time throughout the treatment. In addition, some CAT systems employ bonded resin attachments on teeth to cover the range of motions [63].

## 7. Scope and Advantages

In terms of the scope of CAT, it has a wide therapeutic and functional scope including, aesthetics improvement, oral hygiene, comfort, lack of soft tissue irritation, and periodontal health [63, 64]. Patients who seek CAT have been shown to be motivated mostly by aesthetic considerations [65, 66]. Compared to fixed appliances, CAT has a better impact on oral hygiene due to its removability [67]. Many reports have indicated that CAT reduces plaque accumulation, gingival inflammation, bleeding on probing, pocket depth, and the development of white spot lesions [68–70]. In addition, CAT application was associated with significantly reduced pain, improved functional and psychosocial-related aspects, and lack of soft tissue irritation, compared to the fixed appliances [64, 71]. A recent systematic review by Cardoso et al. showed that, during the initial few days of therapy, orthodontic patients wearing Invisalign appear to experience less pain than those wearing fixed appliances [72].

Interestingly, the CAT group has a substantially shorter chair time, allowing the clinician to treat more patients [69, 73]. This finding showed that time efficiency is better in CAT than fixed appliances. Adult orthodontic patients at risk of periodontitis should consider CAT while planning their treatment. When compared to fixed buccal orthodontic appliances, CAT was shown to be linked with improved periodontal condition and lower levels of periodontopathic bacteria throughout a 12-month trial period [74]. Moreover, Buschang et al. found that CAT is indicated for managing simple malocclusion [63].

## 8. Materials

Regarding the CAT materials, many factors affect the biomechanical characteristics of aligners, such as material thickness, properties of the material, and the accuracy of the aligner and its fitting to the teeth [75]. CAT can be created in a series using a single aligner material; however, in alternative systems, numerous aligner materials may be used in a sequential series or in a repeating cycle of different materials. Some CAT appliances are created using a vacuum, while others are created using pressure [75]. Compared to traditional fixed appliance orthodontic treatment, CAT has various advantages, including improved aesthetics, fewer clinical emergencies, comfort, and a lack of soft tissue irritation [63]. Buschang et al. concluded that CAT is recommended for managing simple malocclusion in a systematic review of the literature. However, the evidence that supports the use of CAT is very weak and heterogeneous [63].

## 9. The Application of CAT in AI and DI

The application of CAT in patients with AI and DI is favorable over the fixed applicants. Nevertheless, its application should be accompanied with caution because of the reduced highest of the crown and lack of undercuts; therefore, aesthetics should be examined and enhanced wherever possible to promote patient compliance [57]. Sockalingam reported a case of a 10-year-old girl with inherited hypoplastic AI [76]. The author mentioned that the posterior teeth were still growing; therefore, they did not recommend using complex and permanent restorations in restoring the defective teeth. Instead, composite restorative material is used to restore defective structures due to its aesthetics and lifespan. It also gives appropriate conservative transitional therapy for AI protection in weak teeth. The damaged surface portions were rebuilt to their original size. In terms of limitations, a single tooth restoration took a prolonged time, and each recovered tooth required additional trimming and polishing.

In another case report, Suchancova and his colleagues reported that the transparent vacuum-formed Essix retainers were used after each orthodontic treatment in a 14-year-old female patient with severe hypocalcified type III of AI holding her teeth in the proper position. They concluded that AI management should be based on a combination of orthodontic and prosthodontic treatments [55]. Similarly, Rajesh et al. used a clear acrylic occlusal splint in a 32-year-old male patient with hypoplastic AI, and their findings also support the need for prosthodontic and orthodontic principles and strategic planning in addition to a multidisciplinary approach in managing a patient with AI [77]. In the study of Khodaeian et al., a 21-year-old male patient with the hypoplastic type of AI in posterior teeth and hypomatured type of AI in anterior teeth was treated by the following strategy: (1) pain control; (2) preventive care and improvement in oral hygiene; (3) caries removal and root canal therapy; (4) orthodontic treatment to manage the anterior and posterior crossbite; (5) periodontal correction of gingival contours in anterior sextant and crown lengthening in posterior sextant by the application of CAT; (6) prosthodontic treatment plan that includes porcelain laminate veneer for maxillary and mandibular incisors and metal-ceramic restoration for other teeth. This strategy highlights the role of CAT in the management of AI in addition to prosthodontic and periodontic treatment [78].  Table 1 summarizes the published case reports regarding the application of CAT in patients with AI.

## 10. Limitations of CAT

The main limitation of CAT is the lack of high-quality supporting evidence, as few published weak investigations (case reports, case series, and expert opinions) have been reported on the prediction of orthodontic tooth movement using CAT. The quantity, quality, and heterogeneity of investigations make it challenging to interpret the findings. As a result, clinicians who want to use CAT must rely on their own clinical experience. Generally, the effect of CAT in controlling the posterior buccolingual inclination is highly predictable; nevertheless, anterior teeth extrusion, rounded teeth rotations, and anterior buccolingual inclination improvement are more difficult to be anticipated [61]. Additionally, extrusion, severe rotation correction, molar uprighting, and extraction gap closure are all known to be more difficult to achieve using aligners [79]. Another limitation of CAT is the possibility of lost or damaged aligners; however, they can be replaced within two weeks [61].

Gay et al. conducted a systematic review that demonstrated that CAT was associated with root resorption at the end of orthodontic treatment, including apical root resorption, with upper and lower incisors being the most affected teeth [80]. A meta-analysis of Zheng and his colleagues found that the rate of root resorption produced by aligners is comparable to that generated by light orthodontic forces [81]. Stability is one of the most critical problems to consider with clear aligners, as with other forms of orthodontic treatment. However, due to the novelty of CAT, there are few retention studies on aligners in the literature, and more research is needed on this point [70, 82]. Recently, Nota and his team showed that, after one month of therapy with clear aligners, individuals might notice a decrease in masseter basal activity, but three months later, this effect seems to return to normal [83].

Furthermore, Damasceno Melo et al. found that CAT can interfere with the patients' speech function three days after the application, leading to noticeable speech difficulties; however, this cannot last for more than 180 days [84]. CAT is more expensive than fixed appliance therapy. Besides, developing a strategy for clear-aligner treatment takes the orthodontist longer than making a plan for fixed appliance therapy [85].

## 11. Conclusion

AI and DI have a significant burden on socializing, function, and comfort; therefore, frequent screening and accurate diagnosis is the cornerstone of managing such conditions. Furthermore, an interdisciplinary combination of orthodontic, prosthodontic, and periodontic treatment has been proven to improve the prognosis of AI and DI. The available evidence regarding the application of CAT in AI is weak and heterogeneous. However, CAT has been reported to be used in each stage of AI treatment. On the other hand, we could not identify any articles regarding the application of CAT in DI. Therefore, we suggest conducting well-designed, longitudinal studies to investigate the role of CAT in patients with AI and/or DI.

## Figures and Tables

**Table 1:** Summary of published case reports regarding the application of CAT in patients with amelogenesis imperfecta.

Study ID	Study design	Patients	Complaints	Treatment plan	Brand	Follow-up	Outcome
Lowe, M 2020	Case report	A 14-year-old male patient	Amelogenesis imperfecta and a severe anterior open bite	Clear aligners and restorative rehabilitation	ClearCorrect	12 months	ClearCorrect aligners overcome the lack of enamel to bond appliances, and their rigidity and style of trimline decrease the need for attachments/engagers. The trimline used by ClearCorrect aligners helps overcome a small generalized tooth size.

Sabandal et al., 2020	Case report	A 16-year old female	Amelogenesis imperfecta type I	Orthodontic and restorative treatment	Invisalign system (Switzerland)	9 years	Decreased discomfort and improvement of the quality of life

Sockalingam. S, 2011	Case report	A 9-year-old Malay girl	Inherited hypoplastic amelogenesis imperfecta	Transparent thermoforming templates	NR	NR	The usage of the templates allowed direct light curing of the composite, accurate reproducibility of the anatomic contours of the defective teeth, reduced chair-side time, and easy contouring and placement of homogenous thickness of composite in otherwise inaccessible sites of the affected teeth

Suchancova et al., 2014	Case report	A 14-year old female	Severe hypocalcified type III of amelogenesis imperfecta	Transparent vacuum-formed Essix retainers	NR	NR	It helps in holding her teeth in the right position

Rajesh et al.,	Case report	A 32-year-old male patient	Hypoplastic amelogenesis imperfecta	Clear acrylic occlusal splint	NR	NR	Treatment improved the patient's aesthetics, function, and comfort

NR: not reported.

## Data Availability

All data are available within the manuscript.
